# Current update on anticancer effects of icariin: A journey of the last ten years

**DOI:** 10.17179/excli2022-4848

**Published:** 2022-04-06

**Authors:** Mukta Gupta, Yachana Mishra, Vijay Mishra, Murtaza M. Tambuwala

**Affiliations:** 1 School of Pharmaceutical Sciences, Lovely Professional University, Phagwara (Punjab)-144411, India; 2 Department of Zoology, Shri Shakti Degree College, Sankhahari, Ghatampur,Kanpur Nagar (UP)-209206, India; 3 School of Pharmacy & Pharmaceutical Sciences, Ulster University, Coleraine, County Londonderry, BT52 1SA, Northern Ireland, United Kingdom

## ⁯

**Figure 1:**
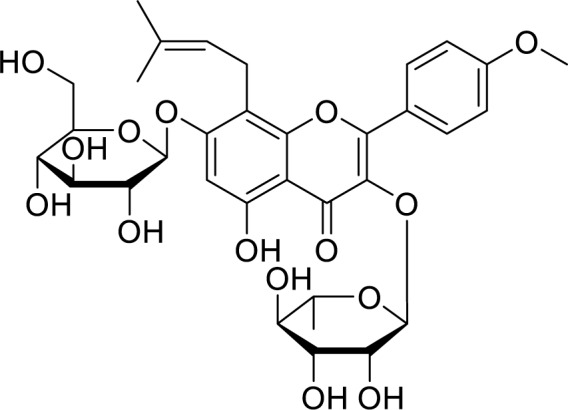
Chemical structure of icariin

Icariin (C_33_H_40_O_15_), a prenylated flavonoid (Figure 1(Fig. 1)), is mainly found in Chinese medicinal herbs of the family *Epimedium*. It is reported for diverse pharmacological activities in different pathological conditions, including inflammation, oxidative stress, cardiac disease, autoimmune system disorders, neurodegeneration, osteoporosis, depression, and cancer (El-Shitany and Eid, 2019[5]; He et al., 2020[10]). Though various *in vivo* and *in vitro* studies have revealed the anticancer effect of icariin, only a few systematic reviews have been published (Tan et al., 2016[23]). As there is a scarcity of studies reflecting the therapeutic role of icariin, the present letter highlights the beneficial effects of icariin in the treatment of different cancer. Further, it will provide a future direction to researchers and hints at developing safe and efficient anticancer drugs (Table 1(Tab. 1); References in Table 1: Alhakamy et al., 2020[3], 2021[2]; Alhakamy, 2021[1]; Cheng et al., 2019[4]; Fan et al., 2016[6]; Fang et al., 2019[7]; Gu et al., 2017[8]; Hao et al., 2019[9]; Huang et al., 2019[11]; Kim et al., 2020[12]; Lei et al., 2020[13]; Li et al., 2010[16], 2014[17], 2015[15], 2021[14]; Ma et al., 2014[18]; Shi et al., 2014[19]; Song et al., 2020[20]; Sun and Zhang, 2021[21]; Sun et al., 2016[22]; Tian et al., 2018[24]; Wang et al., 2010[28], 2015[29], 2017[25], 2019[26], 2020[27]; Wu et al., 2019[30]; Yang and Li, 2020[32]; Yang et al., 2015[31]; Zhang et al., 2013[33], 2014[34]). Ageal mucosa is normal.

**Figure 1:** Chemical structure of icariin

EXCLI Journal 2022;21:680-686 – ISSN 1611-2156
Received: March 19, 2022, accepted: March 23, 2022, published: April 06, 2022

| Type of cancer | Key findings | Reference |
|---|---|---|
| Breast cancer | Song et al. evaluated the antitumor effect of icariin in human breast cancer cell lines (MDA-MB-231) and murine breast cancer cell lines (4T1). A significant reduction of tumor growth and pulmonary metastasis was observed in both MDA-MB-231 and 4T1 cells *via* triggering the process of apoptosis, which is attributed to the activation of nuclear factor-kappa B (NF-κB)/epithelial-mesenchymal transition (EMT) / sirtuin 6 (SIRT-6) pathways. | Song et al., 2020 |
| | The first investigation regarding the beneficial effect of icariin and icaritin in estrogen receptor (ER)-negative breast cancer cell line (SKBr3) was performed by Ma et al. Both icariin and icaritin stimulated apoptosis and inhibited cell proliferation by stimulating G protein-coupled estrogen receptor 1 (GPER1). Also, the phosphorylation of extracellular signal-regulated kinase (ERK) 1 and 2 is enhanced by flavonoids, thus suggesting its role in ER-negative breast cancer. | Ma et al., 2014 |
| | Cheng et al. evaluated the antitumor effect of icariin in tamoxifen (TAM)-resistant breast cancer cell line (MCF-7/TAM). The results indicated that icariin effectively inhibited the growth of MCF-7 and overcame the TAM-induced resistance. It also arrested the cell cycle at $G_0/G_1$ phase, induced apoptosis, and down-regulated the process of autophagy; therefore it could be used in chemo-resistant breast cancer. | Cheng et al., 2019 |
| Ovarian cancer | The role of icariin in inhibiting proliferation and migration of ovarian cancer cell line (SK-OV-3) indicated its potential in treating of ovarian cancer, attributed to suppression of phosphoinositide-3-kinase (PI3K)/AKT signaling pathways. | Wang et al., 2020 |
| | The enhanced cytotoxic effect of icariin phytosomes was investigated in ovarian-3 cancer cells. Results depicted cell cycle arrest at $G_2$/M phase and pre $G_1$ phase followed by elevation of early and late apoptosis. Further, the phytosome formulation disrupted the mitochondrial membrane potential (MMP) and enhanced reactive oxygen species (ROS); therefore it can be further explored to combat tumors. | Alhakamy et al., 2020 |
| | Li et al. evaluated the antiproliferative and apoptotic effect of icariin in human ovarian cancer cells (A2780) by investigating the expression of microRNA-21 (miR-21) and anti-miR-21 target genes. The data indicated that icariin significantly attenuated proliferation and unregulated apoptosis and caspase 3 protein expressions in A2780 cell lines. Moreover, icariin decreased the expression of miR-21 and B-cell lymphoma 2 (Bcl-2) proteins and substantially increased the levels of phosphatase and tensin homolog (PTEN) and reversion-induced cysteine-rich protein with Kazal motifs (RECK), indicating excellent antitumor potential. | Li et al., 2015 |
| | Wang et al. established the antimetastatic effect of icariin against ovarian cancer. The capacity of icariin to suppress the expression of fuse binding protein (FBP) 1 revealed its potential to inhibit proliferation and tumorigenesis through binding to Myc promoter and β-catenin sites. Furthermore, the migration of SKOV3 cells was inhibited by icariin, explaining its role as a therapeutic agent in ovarian cancer therapy. | Wang et al., 2019 |

EXCLI Journal 2022;21:680-686 – ISSN 1611-2156
Received: March 19, 2022, accepted: March 23, 2022, published: April 06, 2022

| Type of cancer | Key findings | Reference |
|---|---|---|
| **Pancreatic cancer** | Alkahamy evaluated the pro-apoptotic activity of icariin-loaded polymeric polylactic-co-glycolic acid (PLGA) with polyethylene glycol (PEG) nanoparticles in pancreatic cancer cell lines. The loss of MMP, generation of ROS, and increase in the number of $G_1$ phase apoptotic cells showed its role in treating pancreatic cancer. | Alhakamy, 2021 |
| | Alkahany et al. prepared an icariin-melittin-loaded bilosome formulation to improve its efficacy against pancreatic cancer cells. Icariin demonstrated an anticancer effect by arresting the cell cycle at S and pre $G_1$ phase by blocking pre-apoptotic behavior. | Alhakamy et al., 2021 |
| **Esophageal cancer** | Fan et al. investigated the antitumor activity of icariin against esophageal squamous cell carcinoma (ESCC) by *in vitro* (human EC109 and TE1 ESCCs cell lines) and *in vivo* (male athymic nude mice) models. The treatment with icariin induced suppression of cell viability, migration and glutathione level and improved apoptosis, ROS, and caspase 9 activity *via* regulating endoplasmic reticulum stress. | Fan et al., 2016 |
| | The inhibitory and antimetastatic effects of icariin were explored in human esophageal carcinoma cells (KYSE70). According to the findings, icariin induced apoptosis *via* ROS-mediated MMP alterations, induction of $G_2$/M phase cell arrest, and by causing migration and invasion of esophageal cells mediated through suppression of P13K/AKT and signal transducer and activator of transcription (STAT) 3 pathways. | Gu et al., 2017 |
| **Lung cancer** | Wu and colleagues observed that icariin induced time- and dose-dependent inhibition of proliferation of lung cancer cells (A549 and H1975) and in the xenograft mouse model, followed by improved apoptosis *via* modulating membrane potential, activation of P13K/AKT signaling pathways, leading to caspase protein activation. However, there was no significant difference in cell cycle arrest by icariin. Hence, it could be an effective therapeutic strategy to treat lung cancer. | Wu et al., 2019 |
| **Colon cancer** | The decrease in human colon carcinoma cell growth and metastasis were caused by icariin in HCT116 cell lines *via* up-regulation of p53 level mediated through suppression of Bcl-2 and activation of Bcl-2 associated X-protein (Bax), thus could be used to treat colon carcinoma. | Tian et al., 2018 |
| | Zhang et al. estimated the down-streaming of resistance of tumor cells towards tumor necrosis factor (TNF) associated apoptosis-inducing ligand (TRAIL) by icariin. The induction of apoptosis by icariin with co-administration by TRAIL was achieved up to 49% in HCT116 colon cancer cells *via* modulating protein expression and stimulation of death receptors. | Kim et al., 2020 |
| **Oral squamous cancer** | Sun et al. investigated the antitumor efficiency of icariin in squamous cell carcinoma cells (SCC9 and Cal 27 cell lines). The improvement in the apoptosis rate and suppression of proliferation were potentiated by icariin *via* caspase 3 and 9 ratio activation. The findings revealed that the tumor-reducing ability of icariin was attributed to the inhibition of NF-κB and P13K/AKT signaling pathways. | Sun and Zhang, 2021 |
| | Lei et al. discovered that icariin protected BALBc mice against squamous cell carcinoma (SCC) induced tumorigenesis. The antiproliferative effect of icariin contributes to the positive impact by mitigating the growth and viability, thus lowering the phosphorylation of p65 and suppressing toll-like receptor (TLR) 4 proteins. | Lei et al., 2020 |

EXCLI Journal 2022;21:680-686 – ISSN 1611-2156
Received: March 19, 2022, accepted: March 23, 2022, published: April 06, 2022

| Type of cancer | Key findings | Reference |
|---|---|---|
| **Colorectal cancer** | Hao et al. investigated the ability of icariin to induce antitumor immunity in C57BL/6 mice as well as B16F10, SMMC-7721, and SPC-A-1 cell lines. The findings indicated that icariin effectively reduced the tumor burden by CD8 T cell infiltration and increased memory T cell frequency, therefore it is justifying its ability to combine with immune checkpoint therapy for cancer treatment. | Hao et al., 2019 |
| | The lowering of radio-resistance associated with a high level of NF-κB was found to be suppressed by icariin in both *in vitro* and *in vivo* analysis. Icariin enhanced the radiation-mediated anti-proliferative effect by arresting the cell cycle at the G2/M phase followed by inhibition of anti-apoptotic genes, thus could be a new approach for radiotherapy in colorectal cancer. | Zhang et al., 2014 |
| | Shi et al. employed a murine model of colorectal cancer cells (CRC) to evaluate the synergistic effect of 5-fluorouracil (5-FU) and icariin in curing colorectal carcinoma. The significant reduction in cell proliferation and stimulation of apoptosis was achieved by combination therapy than individual drugs. Further, the combination of icariin with 5-FU potentiated inhibitory effects on NF-κB; however, further investigations are warranted. | Shi et al., 2014 |
| **Gall bladder cancer** | Zhang et al. evaluated the synergistic antitumor activity of icariin and gemcitabine (GEM) in a xenograft mouse model in female BALB/c and human gallbladder carcinoma cell lines (GBC-SD and SGC-996). The dose-dependent inhibition of proliferation and induction of apoptosis was observed in both cell lines GBC-SD and SGC-996. Also, a reduction in tumor size was observed in mice bearing gall bladder cancer. In addition, icariin promoted NF-κB, Bcl-2, Bcl-xl suppression, activated caspase 3, and arrested cell cycle at the $G_0$-$G_1$ phase. | Zhang et al., 2013 |
| **Cervical cancer** | Huang et al. investigated a protective effect of icariin in cervical cancer HeLa cell line. The results showed enhanced apoptosis through cleavage of caspase 3 and 9 and the phosphatidylinositol 3-kinase-related kinase (mTOR)/PI3K/AKT signaling pathways. Furthermore, up-regulation of Bax level and down-regulation in Bcl-2 expression was observed. | Huang et al., 2019 |
| | Li et al. employed *in vivo* (U14 tumor-bearing mice) and *in vitro* (SiHa cells) models to investigate the antitumor effect of icariin. The dose-dependent inhibition of tumor growth and cell viability was observed after treatment with icariin. Also, icariin altered the composition of gut microbiota. Additionally, icariin significantly reduced the migration rate, invasiveness, and expression of tumor growth factor-beta (TGF-β1), TNF-α, interleukin (IL)-6, IL-17A, and IL-10 in SiHa cells. It down-regulated the expression of Ki67, survivin, Bcl-2, c-Myc and stimulated the increase of P16, P53, Bax levels during *in vivo* experimentation. The antitumor efficacy of icariin was attributed to the modulation of TLR 4/MyD88/NF-κB and Wnt/β-catenin pathways. | Li et al., 2021 |
| **Gastric cancer** | Wang et al. reported the negative effect of icariin on the human gastric cancer cell line (BGC-823). The significant suppression of tumor cell migration and invasion was observed at 50% inhibitory concentration ($IC_{50}$) 128 µg/mL, attributed to the down-regulation of Ras-related C3 botulinum toxin substrate 1 (Rac1) and vasodilator-stimulated phosphoprotein (VASP) expression. | Wang et al., 2010 |

EXCLI Journal 2022;21:680-686 – ISSN 1611-2156
Received: March 19, 2022, accepted: March 23, 2022, published: April 06, 2022

| Type of cancer | Key findings | Reference |
|---|---|---|
| **Skin cancer** | Wang et al. determined the protective effect of icariin against melanoma cancer in B16 cells. The significant inhibition of cell proliferation, migration, and invasion was noticed in icariin-treated B16 cells compared to normal cells; however, the effects were observed in a concentration- and time-dependent manner. Further, icariin promoted melanin secretion and up-regulated tyrosinase activity. The antitumor effect of icariin was due to the enhanced $G_0$ cell cycle arrest and decreased cyclin A and CDK2 levels by suppressing ERK1/p-38/ c-Jun N-terminal kinase (JNK) pathways. | Wang et al., 2017 |
| **Blood cancer** | Wang et al. discovered the synergistic effect of icariin and arsenic trioxide (ATO) in treating acute promyelotic leukemia (APL). The results indicated that icariin enhanced the antitumor activity of ATO by stimulating ROS accumulation in cells, therefore it can be used in combination against APL. | Wang et al., 2015 |
| **Liver cancer** | The anticancer effect of icariin in hepatoma cells was investigated by *in vitro* SMMC 7221 cell line model. The findings revealed that icariin triggered the activation of caspase 9 and Bcl/Bax pathways and induced loss in MMP. In addition, the sustained activation of JNK phosphorylation was promoted by icariin mediated-ROS production. The above findings showed the apoptotic potential of icariin is mediated via the JNK/ROS pathway. | Li et al., 2010 |
| | Li et al. checked out the efficacy of icariin to enhance the antitumor activity of ATO-induced apoptosis *in vivo* and *in vitro* models. The findings indicated that icariin potentiated ATO-induced ROS level and disruption of MMP with inhibition of NF-κB; therefore, it can be used in combination with ATO for better antitumor activity. | Li et al., 2014 |
| **Brain cancer** | A recent study explored the potential of icariin in medulloblastoma treatment in DAOY cells (*in vitro*) and xenograft model (*in vivo*). The icariin restrained cell viability, established apoptosis, suppressed cell migration and invasion in DAOY cells, and inhibited tumor formation in mice, which attributed to the inactivation of janus kinase 1 (JAK) 1/STAT3 and P13/AKT pathways. | Yang and Li, 2020 |
| | The inhibitory effect of icariin against medulloblastoma was determined by Sun and colleagues using DAOY and D341 cell lines and xenograft mouse model. The antiproliferative effect of icariin was attributed to the arrest of the S phase of the cell cycle in medulloblastoma cells and reduced tumor size in mice. The possible mechanism involved in the anticancer activity of icariin was due to the regulation of cyclin A/B1, caspase 3 and 9, Bcl-2, and poly(ADP-ribose) polymerase (PARP) pathways. | Sun et al., 2016 |
| | Yang et al. estimated the synergistic effect of icariin with temozolomide in inducing apoptosis and antiproliferative effects against glioblastoma cell lines. The suppression of the NF-κB enhanced the antitumor effect of temozolomide, providing insight for the use of icariin as a chemotherapeutic agent in clinical settings. | Yang et al., 2015 |
| **Thyroid cancer** | The protective effect of icariin against thyroid cancer was investigated using SW579 and TPC1 cells. The attenuation of cell viability and amelioration of apoptosis was achieved through Bcl/Bax and caspase pathways and the inactivation of AKT and ERK signaling pathways. | Fang et al., 2019 |

Received: March 19, 2022, accepted: March 23, 2022, published: April 06, 2022

## *Conflict of interest*

The authors declare no conflict of interest.

## Conflict of interest

The authors declare no conflict of interest.

## Figures and Tables

**Table 1:**
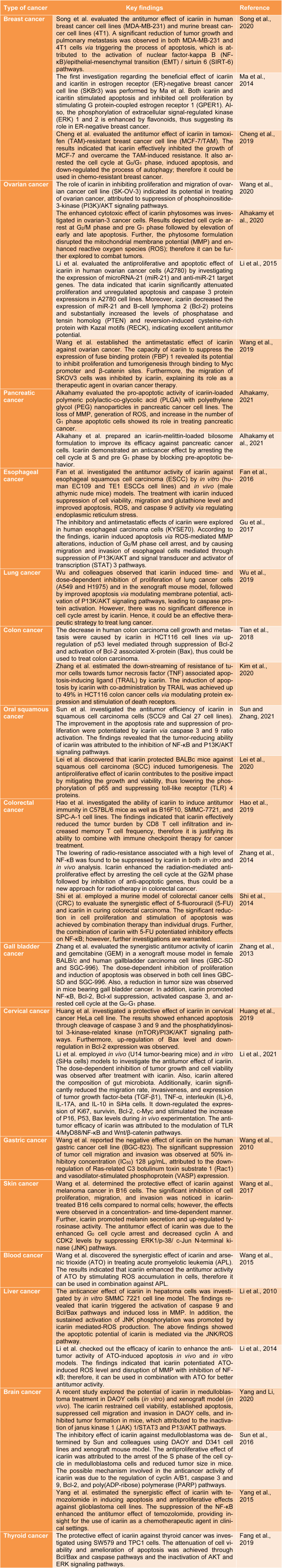
An update on the protective effect of icariin in the treatment of different types of cancer
